# Metabolic Acidosis Is an Independent Risk Factor of Renal Progression in Korean Chronic Kidney Disease Patients: The KNOW-CKD Study Results

**DOI:** 10.3389/fmed.2021.707588

**Published:** 2021-07-29

**Authors:** Hyo Jin Kim, Hyunjin Ryu, Eunjeong Kang, Minjung Kang, Miyeun Han, Sang Heon Song, Joongyub Lee, Ji Yong Jung, Kyu-Beck Lee, Suah Sung, Eun Young Seong, Curie Ahn, Kook-Hwan Oh

**Affiliations:** ^1^Department of Internal Medicine, Pusan National University Hospital, Busan, South Korea; ^2^Biomedical Research Institute, Pusan National University Hospital, Busan, South Korea; ^3^Department of Internal Medicine, Seoul National University Hospital, Seoul, South Korea; ^4^Department of Internal Medicine, Ewha Womans University Seoul Hospital, Ewha Womans University College of Medicine, Seoul, South Korea; ^5^Department of Internal Medicine, Hallym University Hangang Sacred Heart Hospital, Seoul, South Korea; ^6^Department of Preventive Medicine, Seoul National University College of Medicine, Seoul, South Korea; ^7^Department of Internal Medicine, Gachon University Gil Medical Center, Gachon University College of Medicine, Incheon, South Korea; ^8^Department of Internal Medicine, Kangbuk Samsung Hospital, Sungkyunkwan University School of Medicine, Seoul, South Korea; ^9^Department of Internal Medicine, Eulji Medical Center, Eulji University, Seoul, South Korea; ^10^Department of Internal Medicine, National Medical Center, Seoul, South Korea; ^11^Department of Internal Medicine, Seoul National University College of Medicine, Seoul, South Korea

**Keywords:** metabolic acidosis, serum bicarbonate, chronic kidney disease, renal progression, renal function decline

## Abstract

**Background:** We aimed to evaluate serum bicarbonate as a risk factor for renal progression, cardiovascular events, and mortality in Korean CKD patients.

**Methods:** We analyzed 1,808 participants from a Korean CKD cohort whose serum bicarbonate levels were measured at enrollment. Serum bicarbonate levels were categorized as low, lower normal, higher normal, and high (total carbon dioxide <22, 22–26, 26.1–29.9, and ≥30 mmol/L, respectively) groups. Metabolic acidosis was defined as a serum bicarbonate level <22 mmol/L. The primary outcome was renal events defined as doubling of serum creatinine, 50% reduction of eGFR from the baseline values, or development of end-stage kidney disease. The secondary outcome consisted of cardiovascular events and death. In addition, patients whose eGFR values were measured more than three times during the follow-up period were analyzed for eGFR decline. The rapid decline in eGFR was defined as lower than the median value of the eGFR slope.

**Results:** The mean serum bicarbonate level was 25.7 ± 3.7 mmol/L and 240 (13.2%) patients had metabolic acidosis. During the follow-up period of 55.2 ± 24.1 months, 545 (30.9%) patients developed renal events and 187 (10.6%) patients developed a composite of cardiovascular events and death. After adjustment, the low serum bicarbonate group experienced 1.27 times more renal events than the lower normal bicarbonate group [hazard ratio (HR): 1.27; 95% CI: 1.01–1.60, *P* = 0.043]. There was no significant association between the bicarbonate groups and the composite outcome of cardiovascular events and death. The low bicarbonate group showed a significantly rapid decline in eGFR [odds ratio (OR): 2.12; 95% CI: 1.39–3.22, *P* < 0.001] compared to the lower normal bicarbonate group.

**Conclusions:** Metabolic acidosis was significantly associated with increased renal events and a rapid decline in renal function in Korean predialysis CKD patients.

## Introduction

The kidney plays a major role in the maintenance of acid-base balance (1). Therefore, metabolic acidosis is common in cases of decreasing renal function (2). Metabolic acidosis is usually presented as a lowered serum bicarbonate level. In previous studies, when metabolic acidosis was defined as a serum bicarbonate level <22 mmol/L, 2.3–13%, and 19–37% of patients with chronic kidney disease (CKD) stage 3 and 4, respectively, showed metabolic acidosis (3, 4). Metabolic acidosis in CKD has several adverse effects, such as chronic inflammation, bone disease, impaired glucose tolerance, muscle wasting, and possible deleterious consequences of cardiovascular (CV) disease (2, 5).

Metabolic acidosis can also be associated with an accelerated progression of CKD. Acid retention decreases the pH of the renal interstitial and intracellular compartments, causing a rise in the renal levels of angiotensin II and aldosterone, endothelin, ammonia with activation of complement, and proinflammatory cytokines, which are the factors involved in promoting renal fibrosis and injury (6). Previous studies showed that low serum bicarbonate levels were associated with the progression of CKD in outpatients (9% had underlying CKD) (7). Another study showed that low serum bicarbonate levels were associated with the progression of CKD or development of incident CKD in community-living elders (8). The Chronic Renal Insufficiency Cohort (CRIC) study, an observational longitudinal study of US CKD patients, showed that the risk of developing a renal outcome was 3% lower per mEq/L increase in serum bicarbonate (9). Lower serum bicarbonate levels were independently associated with rapid decline in kidney function in non-CKD (10) or CKD patients (11).

Serum bicarbonate levels can also affect CV events and mortality. Both low and high serum bicarbonate levels were associated with increased all-cause mortality in US veterans with moderate and advanced CKD (12). In the CRIC study, metabolic acidosis was correlated with a nominally higher risk of mortality (26%); this result was not statistically significant (13). The risk of heart failure and death was significantly elevated in patients with serum bicarbonate levels >26 mmol/L in the CRIC study (13). There is little data on the long-term clinical outcomes of metabolic acidosis in Korean CKD patients. Therefore, we aimed to investigate the association between metabolic acidosis and renal progression, all-cause mortality, and CV outcomes in CKD patients using data from a large-scale Korean CKD cohort.

## Methods

### Study Design and Population

The KoreaN Cohort Study for Outcome in Patients With Chronic Kidney Disease (KNOW-CKD) was a Korean multicenter prospective cohort study that enrolled subjects with CKD stages 1–5 (predialysis) from nine major university-affiliated hospitals in Korea. The detailed study design and methods of the KNOW-CKD have been described previously (14). Among the 2,238 participants registered in the KNOW-CKD between 2011 and 2016, we included 1,808 subjects whose serum bicarbonate levels were obtained at enrollment. The study protocol was approved by the ethical committee of each participating clinical center and the institutional review boards of Seoul National University Hospital (1104-089-359), Seoul National University Bundang Hospital (B-1106/129-008), Yonsei University Severance Hospital (4-2011-0163)„ Kangbuk Samsung Medical Center (KC11OIMI0441), Seoul St. Mary's Hospital (KC11OIMI0441), Gil Hospital (GIRBA2553), Eulji General Hospital (201105-01), Chonnam National University Hospital (CNUH-2011-092), and Pusan Paik Hospital (11–91) in 2011. All study subjects provided written informed consent. The study protocol was in accordance with the principles of the Declaration of Helsinki.

### Clinical Data Collection and Laboratory Measurements

Baseline demographic characteristics such as age, sex, body mass index (BMI), cause of CKD, smoking, comorbidities, and laboratory data at enrollment were extracted from an electronic data management system (http://www.phactax.org), with assistance from the Division of Data Management at Seoul National University Medical Research Collaborating Center. Patients with a fasting serum glucose ≥126 mg/dL, a history of diabetes mellitus (DM), or those on anti-diabetic medication were considered to have DM. Patients with a systolic blood pressure ≥140 mmHg, a diastolic blood pressure ≥90 mmHg, or a history of hypertension (HTN) were considered to be HTN. Patients considered to have CV disease were those with a history of coronary artery disease, cerebrovascular disease, arrhythmia, congestive heart failure, or peripheral vascular disease. The following laboratory variables were measured using a ≥8-h fasting blood sample at each participating laboratory: total carbon dioxide (TCO_2_), hemoglobin, uric acid, albumin, total cholesterol, C-reactive protein (CRP), phosphorous, calcium, and intact parathyroid hormone. Serum TCO_2_ was considered a surrogate measure of serum bicarbonate (12, 15). Serum creatinine was measured using an isotope dilution mass spectrometry (IDMS)-traceable method (16) at a central laboratory (Lab Genomics, Korea). The estimated glomerular filtration rate (eGFR) was calculated using the Chronic Kidney Disease Epidemiology Collaboration (CKD-EPI) creatinine equation (17). CKD stages 1–5 were defined according to the Kidney Disease: Improving Global Outcomes' guidelines (18). Second voided or random urine samples were immediately sent to a central laboratory to determine the urine creatinine and protein levels. Urinary protein excretion was quantified using urinary protein/creatinine ratio (UPCR, g/g) and urinary albumin/creatinine ratio (UACR, mg/g). Estimated dietary protein intake (eDPI) was calculated using the Maroni–Mitch formula: 6.25 × [urine urea nitrogen (g/day) + 0.03 × body weight (kg)] + proteinuria (g/day) (19), and DPI was calculated by dividing the eDPI by body weight (g/kg/day).

### Study Outcomes

The primary outcome was renal events, defined as eGFR halving or development of end-stage kidney disease. End-stage kidney disease was defined as the initiation of renal replacement therapy, including dialysis or renal transplantation. The secondary composite outcome consisted of CV events and all-cause mortality. Patients were followed until March 2019. The eGFR decline during the follow-up period was also analyzed.

### Statistical Analyses

Categorical variables were evaluated using the χ^2^-test or Fisher's exact test and presented as frequencies and percentages. Continuous variables were analyzed using the analysis of variance or Kruskal–Wallis test. The Kolmogorov–Smirnov test was used to analyze the normality of the distribution of parameters. The results were presented as mean ± standard deviation for variables with normal distribution and the median (interquartile range) for variables with skewed distribution. A log transformation was used to normalize the CRP and proteinuria variables. Participants were categorized into four groups according to their serum bicarbonate levels. Low, lower normal, higher normal, and high TCO_2_ values were defined as <22, 22–26, 26.1–29.9, and ≥30 mmol/L, respectively, considering the guidelines for CKD management, previous reports (7, 13, 20), and normal TCO_2_ range for clinical laboratory. Metabolic acidosis was defined as a TCO_2_ level <22 mmol/L. We used a Cox proportional hazards model with adjustment, including variables that were significant in a univariable analysis or other clinically relevant variables, to analyze the association between the serum bicarbonate levels and study outcomes. The results were presented as hazard ratios (HRs) and 95% confidence intervals (CIs). Patients who were lost to follow-up were censored at the date of the last examination. The rates of renal function decline per year were calculated using the slope of eGFR obtained from a generalized linear mixed model. Only 1,571 (86.9%) patients whose eGFR values were measured more than three times during the follow-up period were included in the eGFR decline analysis. The rapid decline in eGFR was defined as lower than the median value of the eGFR slope. Binary logistic regression analysis was used to identify the risk factors for the rapid decline of renal function. *P* < 0.05 were considered statistically significant. The SPSS statistical software (SPSS version 20.0, IBM Corporation, Armonk, NY, USA) was used for all descriptive and outcome analyses.

## Results

### Baseline Clinical Characteristics of Subjects

The clinical characteristics of the study subjects at enrollment are shown in Table 1. The mean age of the 1,808 patients was 53.6 ± 12.3 years, and 1,111 patients (61.4%) were males. The mean eGFR was 52.8 ± 30.9 mL/min/1.73 m^2^. Patients with DM and HTN comprised 34.8 and 95.8% of the participants, respectively. The mean serum bicarbonate level was 25.7 ± 3.7 mmol/L. When stratified into four groups based on the baseline serum bicarbonate levels, we observed that patients in the low serum bicarbonate group were older (*P* = 0.014) and had a higher prevalence of DM (*P* = 0.011), HTN (*P* = 0.019), and preexisting CV disease (*P* < 0.001) compared to the other three groups. The eGFR (*P* < 0.001) was lower and UPCR (*P* < 0.001) and UACR (*P* < 0.001) were higher in the low serum bicarbonate group. DPI was similar among all bicarbonate groups (*P* = 0.214). Members of the low serum bicarbonate group were prescribed more diuretics than those in the other groups (*P* = 0.001).

**Table 1:** Clinical characteristics of the study subjects at enrollment, stratified by serum bicarbonate concentration.

**Characteristics**	**Total** ** (*N* = 1,808)**	**Serum TCO**_****2****_				***P*-value**
		**Low** ** (<22 mmol/L)** ** (*n* = 240)**	**Lower normal** ** (22–26 mmol/L)** ** (*n* = 760)**	**Higher normal** ** (26.1–29.9 mmol/L)** ** (*n* = 565)**	**High** ** (≥30 mmol/L)** ** (*n* = 243)**	
Age (mean ± SD)	53.6 ± 12.3	54.7 ± 11.7	54.3 ± 12.4	52.4 ± 12.3	52.7 ± 12.4	0.014
Sex, male, *n* (%)	1,111 (61.4)	141 (58.8)	466 (61.3)	345 (61.1)	159 (65.4)	0.492
BMI (kg/m^2^)	24.6 ± 3.4	24.0 ± 3.3	24.7 ± 3.4	24.7 ± 3.6	24.7 ± 3.2	0.024
SBP (mmHg)	127.5 ± 15.6	129.0 ± 19.0	127.0 ± 15.3	127.4 ± 15.1	127.6 ± 13.7	0.389
DM, *n* (%)	627 (34.8)	96 (40.0)	283 (37.2)	179 (31.7)	69 (28.8)	0.011
HTN, *n* (%)	1732 (95.8)	235 (97.9)	735 (96.8)	535 (94.7)	227 (93.4)	0.019
Preexisting CV disease, *n* (%)	302 (16.7)	64 (26.7)	128 (16.8)	82 (14.5)	28 (11.5)	<0.001
CAD, *n* (%)	125 (6.9)	28 (11.8)	56 (7.4)	29 (5.1)	12 (4.9)	0.004
Cerebrovascular ds, *n* (%)	119 (6.6)	20 (8.3)	52 (6.8)	37 (6.5)	10 (4.1)	0.297
HF, *n* (%)	31 (1.7)	11 (4.6)	12 (1.6)	6 (1.1)	2 (0.8)	0.002
Arrhythmia, *n* (%)	41 (2.3)	13 (5.4)	9 (1.2)	14 (2.5)	5 (2.1)	0.002
PVD, *n* (%)	69 (3.8)	15 (6.3)	30 (3.9)	18 (3.2)	6 (2.5)	0.126
Cause of CKD						<0.001
DN, *n* (%)	425 (23.5)	68 (28.3)	213 (28.0)	113 (20.0)	31 (12.8)	
Hypertension, *n* (%)	318 (17.6)	54 (22.5)	135 (17.8)	93 (16.5)	36 (14.8)	
GN, *n* (%)	623 (34.5)	76 (31.7)	250 (32.9)	193 (34.2)	104 (42.8)	
PKD, *n* (%)	326 (18.0)	22 (9.2)	116 (15.3)	133 (23.5)	55 (22.6)	
Others, *n* (%)	116 (6.4)	20 (8.3)	46 (6.1)	33 (5.8)	17 (7.0)	
Smoking status, *n* (%)						0.820
Never	979 (54.1)	125 (52.1)	400 (52.6)	316 (55.9)	138 (56.8)	
Former	554 (30.6)	77 (32.1)	239 (31.4)	165 (29.2)	72 (30.0)	
Current	275 (15.2)	38 (15.8)	121 (15.9)	84 (14.9)	32 (13.2)	
TCO_2_ (mmol/L)	25.7 ± 3.7	19.6 ± 1.9	24.2 ± 1.3	27.9 ± 0.9	31.3 ± 1.4	<0.001
eGFR (mL/min/1.73m^2^)	52.8 ± 30.9	27.7 ± 18.3	45.1 ± 27.7	65.8 ± 29.5	71.9 ± 27.8	<0.001
Hemoglobin (g/dL)	12.8 ± 2.0	11.3 ± 1.7	12.5 ± 2.0	13.5 ± 1.9	13.7 ± 1.8	<0.001
Uric acid (mg/dL)	7.0 ± 1.9	7.5 ± 2.0	7.3 ± 2.0	6.6 ± 1.9	6.6 ± 1.7	<0.001
Albumin (g/dL)	4.2 ± 0.4	4.0 ± 0.4	4.1 ± 0.5	4.3 ± 0.4	4.3 ± 0.4	<0.001
Total cholesterol (mg/dL)	174.3 ± 39.2	164.9 ± 43.8	172.8 ± 39.2	177.6 ± 37.3	180.7 ± 37.1	<0.001
CRP, median, (Q1, Q3) (mg/L)	0.6 (0.2, 1.7)	0.7 (0.4, 2.1)	0.6 (0.2, 1.7)	0.5 (0.2, 1.5)	0.5 (0.2, 1.4)	0.001
Phosphorus (mg/dL)	3.7 ± 0.7	4.1 ± 0.8	3.7 ± 0.7	3.5 ± 0.6	3.6 ± 0.6	<0.001
^*^Corrected Ca (mg/dL)	9.0 ± 0.4	8.8 ± 0.5	9.0 ± 0.4	9.1 ± 0.4	9.1 ± 0.4	<0.001
iPTH, median (Q1, Q3) (pg/mL)	51.0 (33.5, 83.8)	87.3 (52.9, 146.0)	56.5 (36.7, 94.8)	43.0 (30.1, 64.9)	39.1 (27.6, 53.8)	<0.001
UPCR (Q1, Q3) (g/g)	0.49 (0.15, 1.48)	1.08 (0.39, 2.25)	0.58 (0.19, 1.83)	0.34 (0.10, 0.93)	0.27 (0.07, 0.81)	<0.001
UACR (Q1, Q3) (mg/g)	350 (80, 1052)	767 (238, 1481)	420 (121, 1313)	237 (46, 694)	179 (29, 585)	<0.001
DPI (g/kg/day)	0.96 ± 0.30	0.96 ± 0.39	0.94 ± 0.29	0.98 ± 0.28	0.96 ± 0.25	0.214
Medications						
ACEi or ARB, *n* (%)	1553 (85.9)	209 (87.1)	646 (85.0)	484 (85.7)	214 (88.1)	0.625
Diuretics, *n* (%)	568 (0.3)	95 (0.4)	252 (0.3)	144 (0.3)	77 (0.3)	0.001
^†^Phosphate binder, *n* (%)	165 (0.1)	32 (0.1)	76 (0.1)	38 (0.1)	19 (0.1)	0.017

*P-value represents a statistical difference according to the serum bicarbonate groups of each value using the χ^2^-test or Fisher's exact test (categorical variables) or the analysis of variance or Kruskal–Wallis test (continuous variables)*.

* *Corrected Ca (mg/dL) = measured total Ca (mg/dL) + 0.8 × [4 – measured serum albumin (g/dL)]*.

† *Only calcium-based phosphate binders were prescribed to the members of our patient cohort*.

*TCO_2_, total carbon dioxide; SD, standard deviation; BMI, body mass index; SBP, systolic blood pressure; DM, diabetes mellitus; HTN, hypertension; CV, cardiovascular; CAD, coronary artery disease; HF, heart failure; PVD, peripheral vascular disease; CKD, chronic kidney disease; DN, diabetic nephropathy; GN, glomerulonephritis; PKD, polycystic kidney disease; eGFR, estimated glomerular filtration rate as determined by the CKD-EPI creatinine equation; CRP, C-reactive protein; Ca, calcium; iPTH, intact parathyroid hormone; UPCR, urine protein creatinine ratio; UACR, urine albumin creatinine ratio; DPI, dietary protein intake; ACEi, angiotensin converting enzyme inhibitor; ARB, angiotensin II receptor blocker*.

### Distribution of Serum Bicarbonate and Prevalence of Metabolic Acidosis

**Figure 1:**
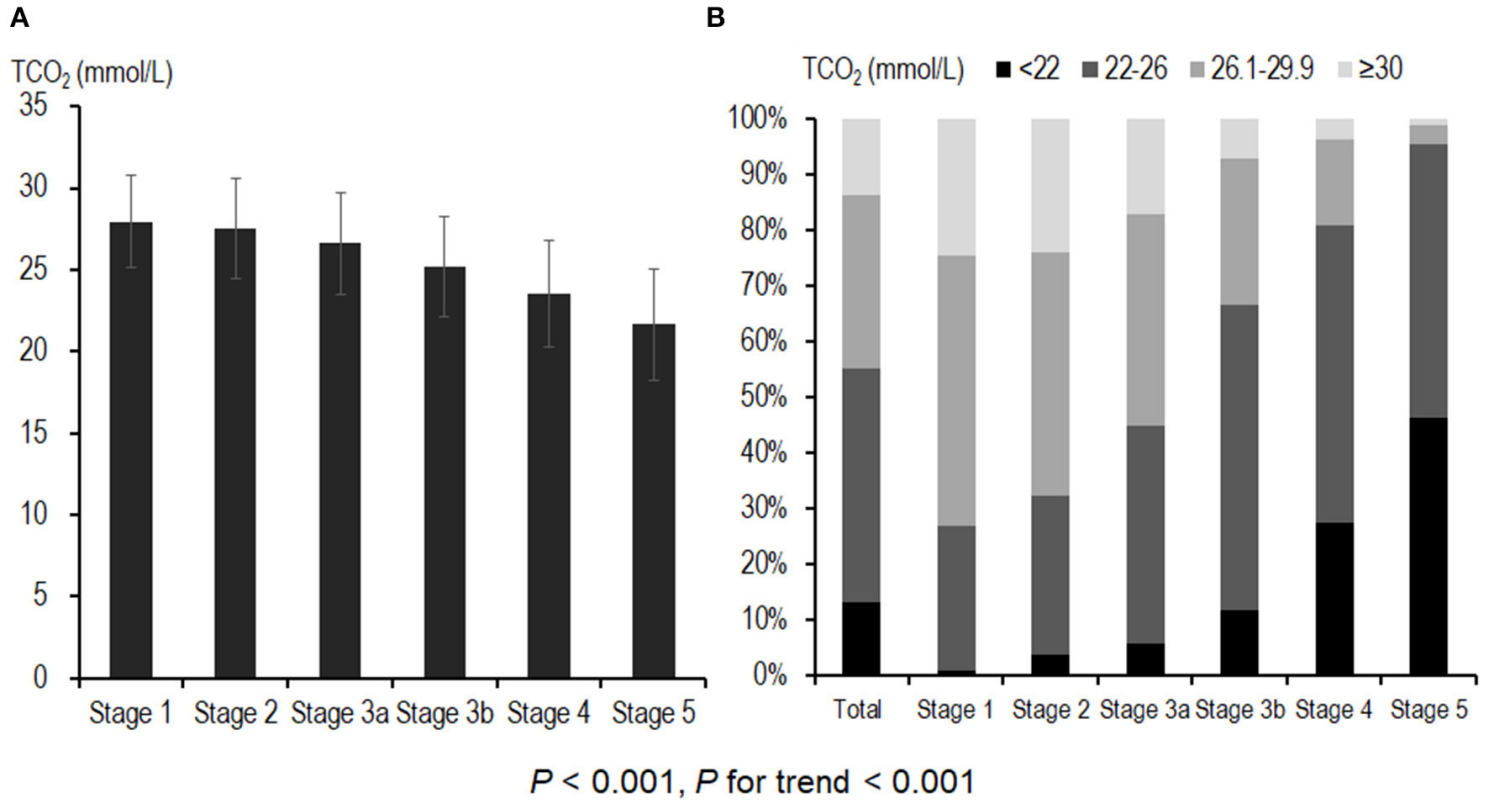
Distribution of serum bicarbonate and prevalence of metabolic acidosis across CKD stages. **(A)** Advanced CKD stages were associated with lower serum bicarbonate levels. A total of 240 (13.2%) patients had metabolic acidosis. **(B)** The prevalence of metabolic acidosis was higher in patients with advanced CKD; 111 (27.6%) and 51 (46.4%) of CKD stage 4 and stage 5 patients, respectively, exhibited metabolic acidosis. CKD, chronic kidney disease; TCO_2_, total carbon dioxide.

Figure 1A shows the distribution of serum bicarbonate across CKD stages. Advanced CKD stages were associated with lower serum bicarbonate levels (*P* < 0.001, *P* for linear trend <0.001; Figure 1A), and 240 (13.2%) patients had metabolic acidosis. The prevalence of metabolic acidosis was higher in patients with advanced CKD (*P* < 0.001, *P* for linear trend <0.001; Figure 1B), and it increased rapidly from CKD stage 4; 1.0, 3.9, 5.8, 12.0, 27.6, and 46.4% patients exhibited metabolic acidosis in CKD stages 1, 2, 3a, 3b, 4, and 5, respectively.

### Serum Bicarbonate and Renal Events

**Figure 2:**
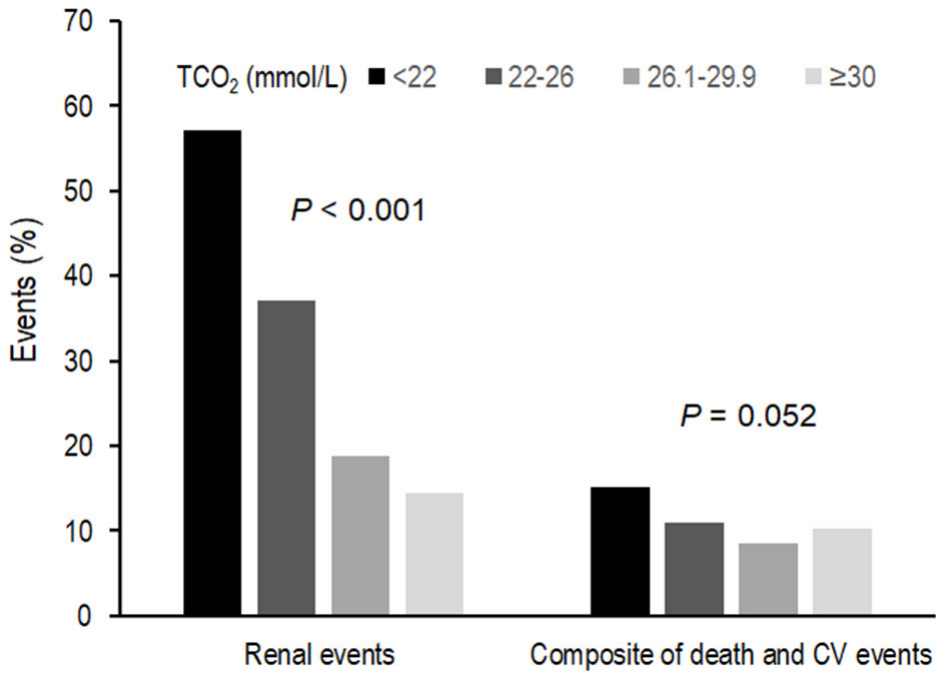
Event rates for renal events and composite of secondary outcomes according to serum bicarbonate groups. Low, lower normal, higher normal, and high TCO_2_ values were defined as <22, 22–26, −26.1 to 29.9, and ≥30 mmol/L, respectively. Patients in the low TCO_2_ group (57.1%) were at a greater risk of developing renal events compared to the other TCO_2_ groups (*P* < 0.001). Patients in the low TCO_2_ group (15.2%) tended to higher composite secondary outcomes (*P* = 0.052). TCO_2_, total carbon dioxide; CV, cardiovascular.

**Figure 3:**
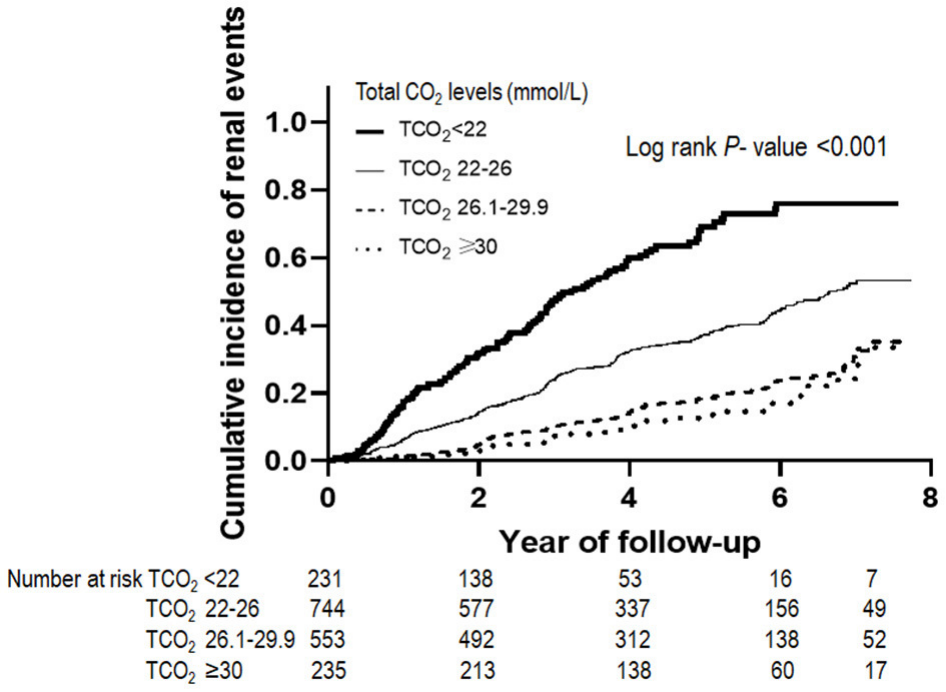
Renal events according to serum bicarbonate groups. The Kaplan–Meier curves show that the low TCO_2_ group had a significantly higher cumulative incidence of renal events (*P* < 0.001). TCO_2_, total carbon dioxide.

Table 2 shows the outcome and event rates according to the serum bicarbonate groups. During the follow-up period of 55.2 ± 24.1 months, 545 (30.9%) patients developed renal events. Patients in the low serum bicarbonate group (57.1%) were at a greater risk for development of renal events compared to the other serum bicarbonate groups (*P* < 0.001; Figure 2). The Kaplan–Meier curves (Figure 3) showed that the low serum bicarbonate group had a significantly higher cumulative incidence of renal events (*P* < 0.001). The multivariable Cox regression analysis showed that the low serum bicarbonate group experienced 1.27 times more renal events than the lower normal serum bicarbonate group (HR: 1.27; 95% CI: 1.01–1.60, *P* = 0.043; Table 3). The higher normal (HR: 0.95; 95% CI: 0.74–1.21, *P* = 0.675) and high serum bicarbonate groups (HR: 0.89; 95% CI: 0.59–1.33, *P* = 0.575) did not experience a significant increase in renal events compared to the lower normal serum bicarbonate group.

**Table 2:** Outcome and event rates according to serum bicarbonate groups.

**Outcomes**	**Overall**	**Serum TCO**_****2****_ **group**				***P*-value**
		**Low** ** (<22 mmol/L)**	**Lower normal** ** (22–26 mmol/L)**	**Higher normal** ** (26.1–29.9 mmol/L)**	**High** ** (≥30 mmol/L)**	
Number of participants	1,808	240	760	565	243	
Renal events, *n* (%)	545 (30.9)	132 (57.1)	275 (37.0)	104 (18.8)	34 (14.5)	<0.001
eGFR halving, *n* (%)	345 (19.6)	59 (25.5)	176 (23.7)	82 (14.8)	28 (11.9)	<0.001
ESKD, *n* (%)	447 (25.4)	126 (54.5)	231 (31.0)	69 (12.5)	21 (8.9)	<0.001
Composite of CV events and death, *n* (%)	187 (10.6)	35 (15.2)	81 (10.9)	47 (8.5)	24 (10.2)	0.052
Non-fatal CV event, *n* (%)	115 (6.5)	20 (8.7)	47 (6.3)	30 (5.4)	18 (7.7)	0.338
Death, *n* (%)	78 (4.4)	16 (6.9)	37 (5.0)	17 (3.1)	8 (3.4)	0.075

*P-value represents a statistical difference according to the serum bicarbonate groups of each event using the χ^2^-test*.

*TCO_2_, total carbon dioxide; eGFR, estimated glomerular filtration rate; ESKD, end-stage kidney disease; CV, cardiovascular*.

**Table 3:** Renal events according to serum bicarbonate concentration.

**Serum bicarbonate**	**Model 1**		**Model 2**		**Model 3**		**Model 4**	
	**HR (95% CI)**	***P*-value**	**HR (95% CI)**	***P*-value**	**HR (95% CI)**	***P*-value**	**HR (95% CI)**	***P*-value**
**Categorical variable**								
Low (<22 mmol/L)	2.35 (1.91, 2.90)	<0.001	2.25 (1.82, 2.78)	<0.001	1.31 (1.05, 1.63)	0.018	1.27 (1.01, 1.60)	0.043
Lower normal (22–26 mmol/L)	Reference	–	Reference	–	Reference	–	Reference	–
Higher normal (26.1–29.9 mmol/L)	0.44 (0.35, 0.55)	<0.001	0.45 (0.36, 0.56)	<0.001	0.96 (0.75, 1.22)	0.760	0.95 (0.74, 1.21)	0.675
High (≥30 mmol/L)	0.33 (0.23, 0.47)	<0.001	0.36 (0.25, 0.51)	<0.001	1.03 (0.71, 1.51)	0.863	0.89 (0.59, 1.33)	0.575
**Continuous variable**								
TCO_2_ (per 1 mmol/L increase)	0.83 (0.81, 0.85)	<0.001	0.84 (0.82, 0.86)	<0.001	0.97 (0.94, 1.00)	0.024	0.96 (0.94, 0.99)	0.013

*Model 1: Unadjusted*.

*Model 2: Adjusted for age, sex, HTN, DM, preexisting CVD, systolic blood pressure, BMI*.

*Model 3: Model 2 + eGFR, log UPCR*.

*Model 4: Model 3 + albumin, total cholesterol, logCRP, ACEi or ARB use, and diuretics use*.

*HR, hazard ratio; CI, confidence interval; TCO_2_, total carbon dioxide; DM, diabetes mellitus; HTN, hypertension; CVD, cardiovascular disease; BMI, body mass index; eGFR, estimated glomerular filtration rate by CKD-EPI creatinine equation; UPCR, urine protein creatinine ratio; CRP, C-reactive protein; ACEi, angiotensin converting enzyme inhibitor; ARB, angiotensin II receptor blocker*.

### Serum Bicarbonate and CV Events and All-Cause Mortality

During the follow-up period, 187 (10.6%) patients developed a composite of CV events and death (Table 2). Patients in the low TCO_2_ group (15.2%) tended to higher composite secondary outcomes (*P* = 0.052; Figure 2). However, in the Cox proportional hazards model after adjustment, there was no significant association between the serum bicarbonate groups and the composite outcome of CV events and death (low vs. lower normal serum bicarbonate group; HR: 1.29; 95% CI: 0.83–2.02, *P* = 0.259; Supplementary Table 1).

### Association of Serum Bicarbonate With Renal Function Decline

**Figure 4:**
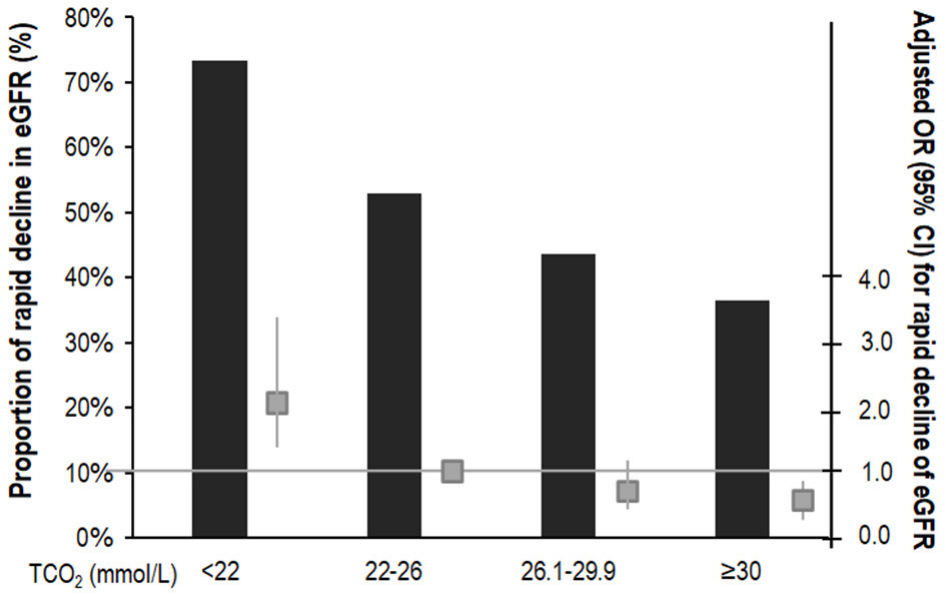
Risk of rapid decline of eGFR according to serum bicarbonate groups. The rapid decline in eGFR was defined as lower than the median value of the eGFR slope. Proportion of the rapid decline of eGFR was higher in the low TCO_2_ group. The multivariable binary logistic regression analysis showed that the low TCO_2_ group had a significantly rapid decline in eGFR (OR: 2.12; 95% CI: 1.39–3.22, *P* < 0.001) compared to the lower normal TCO_2_ group. The column shows a proportion of the rapid decline in eGFR (%). The box plot shows adjusted OR (95% CI) for rapid decline of eGFR. The horizontal solid line represents the reference with an adjusted OR of 1. Adjusted for age, sex, HTN, DM, preexisting CVD, SBP, BMI, eGFR, log UPCR, albumin, total cholesterol, logCRP, ACEi or ARB use, or diuretics. OR, odds ratio; CI, confidence interval; TCO_2_, total carbon dioxide; DM, diabetes mellitus; HTN, hypertension; CVD, cardiovascular disease; BMI, body mass index; eGFR, estimated glomerular filtration rate by CKD-EPI creatinine equation; UPCR, urine protein creatinine ratio; CRP, C-reactive protein; ACEi, angiotensin converting enzyme inhibitor; ARB: angiotensin II receptor blocker.

We analyzed renal function decline as the slope of eGFR for 1,571 patients whose eGFR values were measured more than three times during the follow-up period. Mean eGFR slope was −2.48 ± 2.03 mL/min/1.73 m^2^/year. The eGFR slope according to serum bicarbonate groups was analyzed. The eGFR slope was lower in the low serum bicarbonate group (−2.93 ± 1.61 mL/min/1.73 m^2^/year; Supplementary Figure 1). We categorized the patients into two groups according to the median value of the eGFR slope and rapid decline in eGFR was defined as the group with lower than median eGFR. The proportion of rapid decline in eGFR was higher in the low serum bicarbonate group (Figure 4). The multivariable binary logistic regression analysis revealed that the low serum bicarbonate group showed a significantly rapid decline in eGFR [odds ratio (OR): 2.12; 95% CI: 1.39–3.22, *P* < 0.001; Figure 4] compared to the lower normal serum bicarbonate group.

## Discussion

In the present study, 240 (13.2%) of the 1,808 CKD patients had metabolic acidosis (TCO_2_ <22 mmol/L), and among those with CKD stages 4 and 5, 111 (27.6%) and 51 (46.4%) of patients, respectively, had metabolic acidosis. The incidence of renal events during the follow-up period was higher in patients with metabolic acidosis. Similar to other existing studies, the HR for renal events after adjustment for potential confounders was 1.27 for serum bicarbonate level <22 mmol/L compared to the reference group (serum bicarbonate level 22–26 mmol/L). Patients with metabolic acidosis showed a significantly rapid decline in eGFR. In the present study, there was no significant association between the serum bicarbonate groups and the composite outcome of CV events and death.

Several factors are induced by metabolic acidosis, which cause kidney injury and accelerate the progression of CKD. Kidney damage occurs from acid retention in the interstitial compartment that induces the activation of the renin-angiotensin-aldosterone system and endothelin production (6). In addition, stimulation of complement by increased ammonia production and activation of cytokines also contribute to renal damage. Metabolic acidosis was associated with arterial stiffness in a previous study of our cohort patients (21). Increased arterial stiffness is associated with the risk of renal progression (22); therefore, metabolic acidosis might be associated with CKD progression. Metabolic acidosis is associated with inflammation and oxidative stress, which is associated with renal function deterioration (23, 24); this could also be a mechanistic link between renal injury and metabolic acidosis.

Metabolic acidosis is associated with not only CKD progression but also rapid renal function deterioration. Therefore, proper management is needed to reduce the acid retention. Two modalities that can reduce acid retention include dietary modification to decrease net endogenous acid production or administration of alkali. An experimental study showed that administration of alkali to rats with 5/6 nephrectomy slowed CKD progression and decreased the renal content of endothelin, angiotensin II, and aldosterone (25, 26). A recent randomized trial and other observational studies showed that alkali therapy prevents renal function decline in patients with CKD (27, 28). Large-scale clinical trials are needed to obtain a firm evidence regarding the effectiveness of alkali therapy. Higher serum bicarbonate levels within the normal range were associated with better renal outcomes and survival in the African American Study of Kidney Disease and Hypertension trial (15). Although current guidelines recommend maintaining serum TCO_2_ ≥22 mmol/L, according to previous studies on renal outcomes, mortality, or CV outcomes, the ideal target might be 24–26 mmol/L (29). Therefore, further studies are needed to establish optimal serum bicarbonate concentrations.

Serum bicarbonate <22 mmol/L was associated with increased mortality in US veterans (12); however, metabolic acidosis was not significantly associated with mortality in the CRIC study (13). In addition, an association between metabolic acidosis and increased CV event risk independent of eGFR has not yet been clearly identified. The findings of study outcomes may have been slightly different because of the differences in the characteristics of the study population and the reference range of metabolic acidosis. In the present study, we included patients with early CKD (16.3% with CKD stage 1 and 18.5% with CKD stage 2). Furthermore, our study showed that the incidence rates of mortality and CV outcome were lower than those in the CRIC study (30); hence, the results may differ.

The strength of our study is that we included a large number of predialysis CKD patients. However, this study also has several limitations. First, we could not eliminate the potential residual confounders due to the observational nature of the study. Second, single serum bicarbonate values were used to predict renal events and the composite outcome of mortality or CV events. We did not evaluate the effects of alkali administration (e.g., sodium bicarbonate, sodium lactate, sodium citrate, calcium citrate, etc.) on the incidence of metabolic acidosis and clinical outcomes. There were no patients taking non-calcium-based phosphate binders (e.g., sevelamer carbonate or sevelamer hydrochloride), which affect the acid-base balance. The possibility of dilutional acidosis in metabolic acidosis was excluded because we did not enroll acutely ill patients requiring large volume resuscitation.

## Conclusion

Metabolic acidosis, defined as a serum bicarbonate level <22 mmol/L, was significantly associated with increased renal events and rapid decline of renal function in this cohort of Korean predialysis CKD patients.

## Data Availability Statement

The original contributions presented in the study are included in the article/Supplementary Material, further inquiries can be directed to the corresponding author/s.

## Ethics Statement

The studies involving human participants were reviewed and approved by the study protocol was approved by the ethical committee of each participating clinical center and the institutional review boards of Seoul National University Hospital (1104-089-359), Seoul National University Bundang Hospital (B-1106/129-008), Yonsei University Severance Hospital (4-2011-0163), Kangbuk Samsung Medical Center (2011-01-076), Seoul St. Mary's Hospital (KC11OIMI0441), Gil Hospital (GIRBA2553), Eulji General Hospital (201105-01), Chonnam National University Hospital (CNUH-2011-092), and Pusan Paik Hospital (11–91). The patients/participants provided their written informed consent to participate in this study.

## Author Contributions

HJK and K-HO were involved with the conception and design of the study. HJK, HR, EK, MK, MH, JYJ, K-BL, SS, CA, and K-HO were involved with patient data collection and acquisition. HJK, SHS, JL, EYS, and K-HO performed the analysis and interpretation of data. Article draft and revision were carried out by HJK and K-HO. All authors approved the final manuscript.

## Conflict of Interest

The authors declare that the research was conducted in the absence of any commercial or financial relationships that could be construed as a potential conflict of interest.

## Publisher's Note

All claims expressed in this article are solely those of the authors and do not necessarily represent those of their affiliated organizations, or those of the publisher, the editors and the reviewers. Any product that may be evaluated in this article, or claim that may be made by its manufacturer, is not guaranteed or endorsed by the publisher.
