# Supplementary material for: Metabolic Acidosis Is an Independent Risk Factor of Renal Progression in Korean Chronic Kidney Disease Patients: The KNOW-CKD Study Results
Source: Front Med (Lausanne). 2021 Jul 29;8:707588. doi: 10.3389/fmed.2021.707588 (PMC8358180; doi:10.3389/fmed.2021.707588)
Supplement: Supplementary file 1 [file Data_Sheet_1.docx]

Supplementary Material

**Table S1. Composite secondary outcomes according to serum bicarbonate concentration**

|  | **Model 1** | | **Model 2** | | **Model 3** | | **Model 4** | |
| --- | --- | --- | --- | --- | --- | --- | --- | --- |
| **Serum bicarbonate** | **HR (95% CI)** | ***P*-value** | **HR (95% CI)** | ***P*-value** | **HR (95% CI)** | ***P*-value** | **HR (95% CI)** | ***P*-value** |
| **Categorical variable** |  |  |  |  |  |  |  |  |
| **Low**  (<22 mmol/L) | 1.56 (1.05, 2.32) | 0.029 | 1.41 (0.94, 2.10) | 0.093 | 1.38 (0.91, 2.10) | 0.134 | 1.29 (0.83, 2.02) | 0.259 |
| **Lower normal**  (22-26 mmol/L) | Reference | - | Reference | - | Reference | - | Reference | - |
| **Higher normal**  (26.1-29.9 mmol/L) | 0.79 (0.55, 1.13) | 0.199 | 0.93 (0.64, 1.33) | 0.676 | 1.02 (0.70, 1.50) | 0.917 | 1.14 (0.76, 1.69) | 0.532 |
| **High**  (≥30 mmol/L) | 0.96 (0.61, 1.52) | 0.877 | 1.27 (0.80, 2.03) | 0.312 | 1.40 (0.85, 2.32) | 0.187 | 1.25 (0.72, 2.16) | 0.426 |
| **Continuous variable** |  |  |  |  |  |  |  |  |
| **TCO_2_**  **(per 1 mmol/L increase)** | 0.94 (0.91, 0.98) | 0.004 | 0.98 (0.94, 1.02) | 0.315 | 0.99 (0.94, 1.04) | 0.619 | 0.99 (0.94, 1.04) | 0.741 |
| Composite secondary outcomes consist cardiovascular events and all-cause mortality.  Model 1: Unadjusted  Model 2: Adjusted for age, sex, HTN, DM, preexisting CVD, systolic blood pressure, BMI  Model 3: Model 2 + eGFR, log UPCR  Model 4: Model 3 + albumin, total cholesterol, logCRP, ACEi or ARB use, and diuretics use | | | | | | | | |

HR: hazard ratio; CI: confidence interval; TCO_2_: total carbon dioxide; DM: diabetes mellitus; HTN: hypertension; CVD: cardiovascular disease; BMI: body mass index; eGFR: estimated glomerular filtration rate by CKD-EPI creatinine equation; UPCR: urine protein creatinine ratio; CRP: C-reactive protein; ACEi: angiotensin converting enzyme inhibitor; ARB: angiotensin II receptor blocker

**Figure S1. The eGFR slope according to serum bicarbonate groups**

| **Serum TCO_2_** | **eGFR slope** |
| --- | --- |
| **Low** (<22 mmol/L) | -2.93 ± 1.61 |
| **Lower normal** (22-26 mmol/L) | -2.60 ± 1.97 |
| **Higher normal** (26.1-29.9 mmol/L) | -2.32 ± 2.18 |
| **High** (≥30 mmol/L) | -2.13 ± 2.11 |


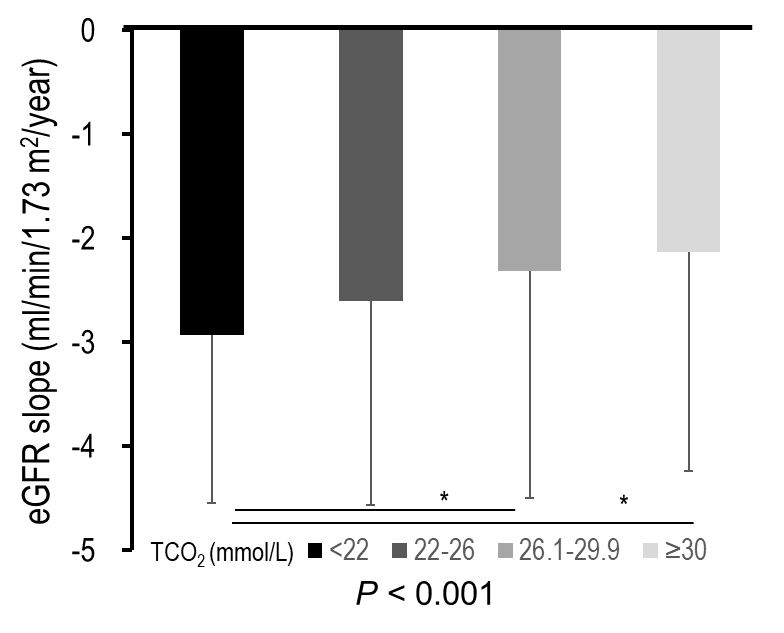


The eGFR slope was analyzed in 1,571 patients with eGFR measured more than three times during the follow-up period.

**P* < 0.001; eGFR: estimated glomerular filtration rate by CKD-EPI creatinine equation; TCO_2_: total CO_2_
